# An Insulin-Like Modular Basis for the Evolution of Glucose Transporters (GLUT) with Implications for Diabetes

**Published:** 2007-10-15

**Authors:** Robert Root-Bernstein

**Affiliations:** Department of Physiology, 2174 Biomedical and Physical Sciences Building, Michigan State University, East Lansing, MI 48824 U.S.A

## Abstract

Glucose transporters (GLUT) are twelve-transmembrane spanning proteins that contain two pores capable of transporting glucose and dehydroascorbate in and out of cells. The mechanism by which transport is effected is unknown. An evolutionarily-based hypothesis for the mechanism of glucose transport is presented here based on reports that insulin has multiple binding sites for glucose. It is proposed that insulin-like peptides were incorporated as modular elements into transmembrane proteins during evolution, resulting in glucose transporting capacity. Homology searching reveals that all GLUT contain multiple copies of insulin-like regions. These regions map onto a model of GLUT in positions that define the glucose transport cores. This observation provides a mechanism for glucose transport involving the diffusion of glucose from one insulin-like glucose-binding region to another. It also suggests a mechanism by which glucose disregulation may occur in both type 1 and type 2 diabetes: insulin rapidly self-glycates under hyperglycemic conditions. Insulin-like regions of GLUT may also self-glycate rapidly, thereby interfering with transport of glucose into cells and disabling GLUT sensing of blood glucose levels. All aspects of the hypothesis are experimentally testable.

## Introduction

Root-Bernstein and Dillon have suggested that molecular complementarity acts as a selective pressure that directs evolution in particular directions by linking molecular structure and function. In essence, those molecules that bind to one another are more likely to survive degradative processes and so become the materials upon which living systems build. Further, the complexes that such pairs of molecules will form will have emergent properties that their individual components do not, thereby providing a constant source of novelty upon which further complexity can be built. (Root-Bernstein and Dillon, 1997; Hunding et al. 2006) Root-Bernstein recently applied this theory to the elucidation of insulin receptor evolution. Insulin both self-aggregates and binds to glucagon, making insulin self-complementary and complementary to glucagon. (Root-Bernstein and Dobblestein, 1999) Notably, the insulin receptor has multiple insulin-like and glucagon-like sequences located in regions associated with insulin binding, and these regions are highly conserved throughout evolution (Root-Bernstein, 2004; Root-Bernstein, 2005).

Another interesting aspect of insulin behavior is that glucose binds directly to it, suggesting that glucose and insulin were linked functionally very early in evolution. Various physicochemical methods (Anzenbacher and Kalous, 1975; Kalous and Anzenbacher, 1979), x-ray crystallography (Yu and Caspar, 1998), and molecular modeling programs (Zoete, Meuwly and Karplus, 2004) have demonstrated that eight D-glucose binding sites exist on the insulin molecule, two with a binding constant of about 1 × 10^−3^ M and six with a binding constant of about 6 × 10^−2^ M. This means that at normal blood glucose of 5 × 10^−3^ M, the two higher affinity sites are likely to be occupied, while the lower affinity sites are likely to be sensitive only to hyperglycemic conditions. One can hypothesize that prior to the origins of insulin receptors and glucose transport proteins, an insulin-like molecule may have carried glucose into cells itself. In addition, in the presence of glucose, insulin is rapidly and non-enzymatically glycated, which may have a variety of effects to be discussed below.

The binding of glucose to insulin, combined with the molecular complementarity theory of evolution, suggested that perhaps glucose transporters (GLUT) might have evolved from an insulin-like precursor that could also bind glucose. If that were the case, then one would expect to find modular insulin-like regions in the transporter core of GLUT but not at the transporter cores of other sugar transporters. This paper reports that this prediction is correct. It describes how such a structure results in functional glucose transport and how glycation of the insulin-like regions in GLUT might result in insulin resistance and inability to transport glucose efficiently.

## Methods

Sequence similarities were determined between the human insulin precursor (SwissProt ID P01308) and all human GLUT precursor sequences available on the SWISSPROT database as of November 2006 (GTR1 P11166; GTR2 P11168; GTR3 11169; GTR4 P14672; GTR5 P22732; GTR6 Q9UGQ3; GTR8 Q9NY64; GTR9 Q9NRM0; GTR10 O95528; GTR11 Q9BYW1; GTR14 Q8TDB8). A further set of similarity searches was carried out in November 2006 involving the sodium/glucose cotransporters 1, 2 and 3 (P13866, P31639, and Q9NY91), using as control sequences the sodium dependent phosphate transporter proteins 1, 2A, 2B, 2C, 3, and 4 (Q14916, Q06495, O95436, Q8N130, O00624, O00476), the sodium dependent phosphate transporter 1 (Q8WUM9), the yeast mannose transporter (P40107), and the arabidopsis mannose transporter (Q941R4).

Similarities were determined using LALIGN (Huang and Miller, 1991). The best 20 similarities were found using default parameters for scoring (penalty for the first residue in a gap, −14; penalty for each additional residue in a gap, −4). Sequences were deemed to be significantly homologous only if they had at least fifty percent identity over a sequence of at least ten amino acids or a raw score (E) of at least 35. Ten amino acid-long sequences were chosen because ten amino acid stretches are approximately the length recognized by T-cell receptors and are therefore of some biological significance (Rudensky et al. 1991) and because in previous studies, it was determined using LALIGN that the probability of a hormone such as insulin or glucagon having five identical amino acids within any stretch of ten amino acids in randomly selected receptors or transporters is six percent (p = 0.06) (Root-Bernstein, 2002). A secondary estimation of the significance of the similarities was also used, involving raw scores. Raw scores (E) are used by LALIGN to derive a function that calculates the probability that an alignment with such a score is likely to occur if any peptide of an equivalent length were used to search 10,000 proteins of equivalent length to the receptor or transporter sequence. Raw scores of 35 or above in this study always corresponded to probabilities of less than 1 in 20 that such alignments occur by chance (p < 0.05). Raw scores above 50 generally corresponded to probabilities of less than 1 in 100 that such alignments occur by chance (p < 0.01). The vast majority of the sequences described in the tables below satisfy both sets of criteria for significance.

It must be emphasized that the probabilities presented above correspond to that of finding a *single* homology between a peptide hormone and a receptor satisfying one or the other criteria. The probability that *more than one* such significant homology would occur within a single receptor or transporter is a multiplicative function of the number of significant sequences found. The probability of finding four such similarities within a single transporter sequence is approximately (0.05)^4^ or about 6 × 10^−6^ and the probability of finding seven such significant similarities is about 10^−11^. All of the GLUT transporters have between four and twelve significant homologies so that the probability that the results presented here have occurred by chance are extremely small.

The sequences of GLUT transporters that had high homology with insulin were mapped onto the model of the GLUT transporter developed by Zuniga et al. (Zuniga et al. 2001) using the amino acid identifiers in their model.

## Results

The results of the homology study are displayed in Tables 1 through 14. As can be seen by inspection, every human GLUT sequence currently available on SwissProt has multiple homologies with insulin satisfying the criteria described above (Tables 1–11). Particularly notable are GLUT 1, GLUT 4, GLUT 6, GLUT 10, and GLUT 11. GLUT 1 has eight significant homologies; GLUT 4 has ten; GLUT 6 and 10 have seven, and GLUT 11 has eleven. Most strikingly, GLUT 11 has one homology that represents virtually the entire unprocessed insulin chain. Many significant similarities were also found between the sodium/glucose cotransporter proteins and insulin (Tables 12–14).

No significant similarities at all were found between insulin and the sodium dependent phosphate transport proteins, the sodium dependent phosphate transporter, or mannose transporters (data not shown, since there is nothing to show). These results are in accord with our previous observation that insulin-like regions are extremely rare in randomly selected receptors and enzymes (Root-Bernstein, 2002) It is also interesting to note that mannose transporters lack significant homologies to the QLSQQLS, and QLG sequences that have been identified through amino acid substitution and modeling as probable glucose recognition regions in GLUT (Seatter et al. 1998; Olsowski et al. 2000, Salas-Burgos, 2004).

On the other hand, insulin itself contains several regions that have the QLS and QLG motifs associated with insulin binding—although these are sometimes in the reversed order—and these motifs occur in regions with significant homologies to GLUT transport cores (see underlines in Tables 1–11). Thus, the insulin-like homologies reported here correspond to putative glucose binding regions identified by previous investigators of GLUT structure (Seatter et al. 1998; Olsowski et al. 2000, Salas-Burgos, 2004; Hruz and Mueckler, 2001; Zuniga et al. 2001).

**Figure 1.**
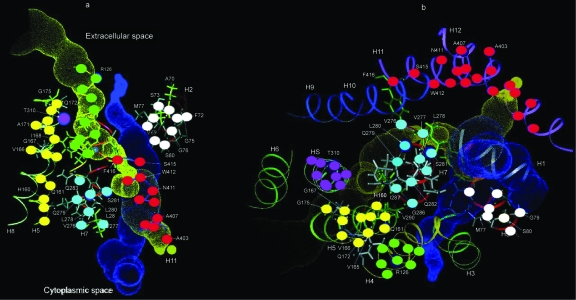
Regions of shared homology between GLUT 1, GLUT 4 and insulin (color coded in TABLE 15) mapped onto Zuniga et al.’s (2001) model of the GLUT transport core.

In order to demonstrate the location of these insulin-like regions in the GLUT structure, the homologies listed in Tables 1–11 (the GLUT sequences) have been mapped onto Zuniga et al.’s model of GLUT transport cores (Zuniga et al. 2001). To simplify the visualization of the placement of the various homologies, only those shared by GLUT 1 and GLUT 4 (arguably two of the most important of the GLUTs) were used; these were reformatted (Table 15) to display their commonalities; and these common areas were then mapped onto Zuniga’s model of the transport cores by means of color coded dots (Figure 1). Figure 1 demonstrates that many of the insulin-like regions that are common among the GLUTs can be assigned to regions of the GLUTs thought to define the transporter core itself. Notably, these insulin-like regions are found to be in the transporter cores of all models of GLUT (e.g. Seatter et al. 1998; Olsowski et al. 2000, Salas-Burgos, 2004; Hruz and Mueckler, 2001). Moreover, all of the amino acids thus far identified as contributing to glucose recognition and binding through mutagenesis studies fall into these insulin-like regions or immediately adjacent to them, including GLUT 1 residues W65, S66, T137, V140, QLS 161–163, V165, QLS 279–285, T310, N317, T321, R333, P387, and W388 (Seatter et al. 1998; Olsowski et al. 2000, Salas-Burgos, 2004; Hruz and Mueckler, 2001; Zuniga et al. 2001).

These data therefore show that the transport cores of GLUT, as defined by current experimental and modeling data, are composed of repeating subunits, or modules, based on an insulin-like sequence. It is further reasonable to propose that since insulin has multiple binding sites for glucose, these insulin-like modular regions are the basis for GLUT binding and transport of glucose.

## Discussion

The fact that glucose binds to insulin and that insulin-like regions make up the transport cores of GLUT proteins suggests an obvious mechanism by which glucose can be transported through these proteins. Glucose would be attracted from extracellular plasma to the glucose-binding sites on the insulin-like regions in the transport core. Simple diffusion from one site to another within the transport core would carry the glucose from the extracellular to the intracellular side of the transporter as long as the concentration of glucose was higher outside the cell than inside. Because the proposed mechanism is passive, diffusion could also occur from the inside of the cell out if the concentration of glucose were higher within the cell than outside it. If the binding affinities were essentially equal from one site within the transport core to the next, then transport would occur with equal velocity in either direction. If the bindings progress either toward greater affinity from outside in, or vice versa, then the velocity of diffusion will differ depending on whether the glucose is diffusing into or out of the cell. The difference in rate will depend on the difference in affinities across the transporter. Differential affinities might be of value in regulating rates of glucose flow.

The fact that multiple insulin-like regions occur within the transport core should not come as a great surprise in terms of the way in which most proteins are thought to have evolved. An ever-increasing body of literature suggests that large proteins are often conglomerates of repeating subunits or modules (e.g. Patthy, 1999; Liu and Rost, 2004; Bornberg-Bauer et al. 2005). Thus, the structure proposed here for GLUT proteins may be seen as just one of many known modular protein structures. What is unusual about this example of modularity is that it may have been based on complementarity between a pair of simple molecules (insulin and glucose) that dictated selection for the specific utility and function of the modules during the evolutionary process itself (Root-Bernstein and Dillon, 1997). Since this is also the case in the insulin receptor, one may speculate that such complementarity will be found in other receptor and transporter proteins as well.

The hypothesis that GLUT evolved from the stringing together of insulin-like modules capable of binding glucose makes a number of novel, experimentally testable predictions. Synthetic peptides derived from the regions of insulin-like homology listed in the Tables above should bind glucose with approximately the affinity previously found for insulin-glucose binding sites. For reasons described above regarding the rate of flow of glucose through the transporter, it will be interesting and important to determine whether all of the binding sites are more or less similar in their glucose affinity, or whether they increase in one direction or the other.

A second prediction of the hypothesis is that these insulin-like regions of GLUT should glycate rapidly (hours to days) in hyperglycemic conditions. It has been shown by several groups of investigators that insulin in the presence of either unusually high concentrations of glucose, or normal glucose concentrations for longer periods of time, will auto-glycate. (Farah et al. 2005; McKillop et al. 2003; Abdel-Wahab et al. 2002; Boyd et al. 2000; O’Harte et al. 2000; Abdel, Wahab et al. 1997a, b; O’Harte et al. 1996; Dollhofer and Wieland, 1979). This auto-glycation occurs on both N- and O-groups of amino acids; is non-enzymatic; and its effect on insulin is to decrease its activity. In contrast to the very slow (weeks to months) non-enzymatic glycation of most proteins (Schalkwijk, Stehouwer and van Hinsbergh, 2004; Vlassara and Palace, 2002; Baynes, 2001), the glycation of insulin is very fast, beginning within hours and reaching a maximum within 24 to 36 hours in hyperglycemic conditions. (Farah et al. 2005; McKillop et al. 2003; Abdel-Wahab et al. 2002; Boyd et al. 2000; O’Harte et al. 2000; Abdel, Wahab et al. 1997a, b; O’Harte et al. 1996; Dollhofer and Wieland, 1979) One would expect the same rapid glycation to occur on the insulin-like regions of GLUT. This prediction can be tested both *in vitro* using insulin-like peptide regions derived from GLUT, or under *in vivo* conditions, in which the GLUT are isolated and analyzed for the presence of glycation products within specific regions of the transport core. If glycation is present under in vivo conditions, it would further be predicted that glucose transport would decrease inversely to increasing GLUT glycation.

A third test of the hypothesis stems from the fact that GLUT also transport dehydroascorbate (DHA) (reviewed in Root-Bernstein, Busik and Henry, 2002). It can be predicted, therefore, that insulin and the insulin-like regions of GLUT should bind DHA with affinities approximating those which they bind glucose. Moreover, DHA binding may significantly retard glycation, which can be tested in both the *in vitro* and *in vivo* conditions just specified above. This prediction may be of clinical significance as well (see below).

A final test of the hypothesis is bioinformatic. All glucose transporters in all species should contain insulin-like sequences in their transport cores that are very highly conserved compared to other regions of the transporters. One of the interesting questions that arises evolutionarily is therefore whether insulin evolved prior to glucose transporters, parallel to them, or from some precursor of the glucose transporters. Currently available data do not permit this problem to be resolved. Determining the structural relationships of insulins and GLUT derived from a very wide range of organisms may therefore shed important light on the molecular origins of glucose regulation and, in turn, new targets for the development of novel therapeutic regimens for diabetes.

## Clinical Implications

The presence of insulin-like sequences in the GLUT has several pratical implications for understanding the consequences of hyperglycemia in both type 1 and type 2 diabetes. Perhaps the most important follows from the possibility that the insulin-like regions of GLUT glycate rapidly under hyperglycemic conditions. (Parenthetically, one might expect fructose glycation of GLUT 2 and GLUT 5 since these transport fructose as well as glucose [Schalkwijk, Stehouwer and van Hinsbergh, 2004]). The glycation of binding sites for glucose transport would be predicted to interfere with glucose transport by blocking the transport cores, thus exacerbating hyperglycemia. The greater the hyperglycemia, the greater the glycation, and the greater the percentage of GLUTs that would malfunction. A positive feedback system would be set up that is self-destructive. The paradoxical result would be that the greater the hyperglycemia, the less glucose would be transported into cells. Cells, starved for a ready energy source, would have to metabolize protein, resulting in the ketosis associated with hyperglycemia. In children with diabetes, the result of such refractory glucose transport would be to inhibit critical developmental processes that are glucose-dependent, such as bone growth (Mobasheri et al. 2002) and insulin-dependent neuron growth (Whitmer, 2007), resulting in long term deficits.

Two additional effects of hyperglycemic glycation might also result. First, if GLUT and sodium-dependent glucose transporters are critical sensors for blood glucose concentration as is currently thought (Freeman et al. 2006; Uldry and Thorens, 2004; Bogacka et al. 2004; Ferber et al. 1994; Leloup et al. 1994), then glucose regulation might be impaired because glucose sensing would be impaired not only in somatic cells but in the brain. Various types of neurons act as glucose sensors in the brain, helping to regulate glucose homeostasis (Parton et al. 2007), so that blocking glucose transport in neurons would also exacerbate glucose disregulation. Secondly, if the same rapid glycation reaction occurs on insulin-like regions of the insulin receptor (Root-Bernstein, 2002; Root-Bernstein, 2005), then insulin activation of its receptor may be impaired, resulting in the insulin-resistance that is associated with diabetes. The combination of these two malfunctions would be an inability for the glucose-regulatory system to sense or respond to its normal regulatory cues.

Understanding the evolutionary structure-function relationship of insulin and GLUT may also provide hints about a novel therapeutic approach to treating auto-glycation. Since GLUT transport dehydroascorbate (DHA), it is possible that serum ascorbate or DHA levels may be a critical factor in preventing (the assumed) GLUT auto-glycation. In essence, higher levels of DHA (which can be generated by higher levels of dietary or transfused ascorbate or DHA itself), will compete with glucose for the GLUT transport sites; DHA competition for glucose binding sites on insulin-like regions of GLUT may decrease auto-glycation of GLUT (as well as on insulin and the insulin receptor), thereby helping to prevent some of the deleterious effects of hyperglycemia. This possibility is also testable by determining rates of auto-glycation of insulin and or insulin-like regions in GLUT in the presence and absence of various concentrations of DHA and ascorbic acid. The likelihood that this approach will be successful is indicated by a report by Abdel-Wahab et al. (2002) that ascorbic acid supplementation decreases insulin glycation in obese hyperglycemic mice. Clearly, these results need to be extended to determine rates of glycation of GLUT and insulin receptors in the presence of ascorbate and DHA.

Finally, the same factors just discussed in terms of diabetes may have relevance for understanding some aspects of normal aging as well. The rate of protein turn-over, particularly of GLUT, is well-documented to decrease with increasing age (Mooradian and Shah, 1997). This means that as a person ages, GLUT will be exposed for longer times to normal glucose serum concentrations. In addition, ascorbate (and thus DHA) deficiency increases with age (Junquiera et al. 2004) The result of prolonged exposure of GLUT to glucose and decreased antioxidant concentrations would be increased GLUT glycation, resulting in body-wide decreased sugar transport and decreased glucose sensitivity. These are early symptoms of metabolic syndrome, a pre-diabetic state also known to increase in prevalence with aging (Rodriguez et al. 2007; Yaffe, 2007). Indeed, the formation of advanced glycation endproducts is a major problem not only in diabetes, but in dementias as well (Vlassara and Palace, 2002; Baynes, 2001). Perhaps these syndromes, too, might be responsive to increased DHA intake.

## Summary

In sum, I have proposed an evolutionary basis for GLUT structure based on a modular units originating in insulin-glucose binding. The model that results suggests various experimentally testable implications including a mechanism for GLUT transport of glucose via a series of insulin-like sequences that form the GLUT transport cores. The proposed structure also suggests the possibility that GLUT are auto-glycated under hyperglycemic conditions, resulting in defective glucose transport and regulation, and it proposes that dehydroascorbate (DHA) may prevent such auto-glycation by direct competition for the glucose binding sites. Thus, a simple modular approach to GLUT evolution based on small molecule complementarity may provide basic insights into the structure and function of a major protein and its modifications in disease states.

## Figures and Tables

**Table 1.**
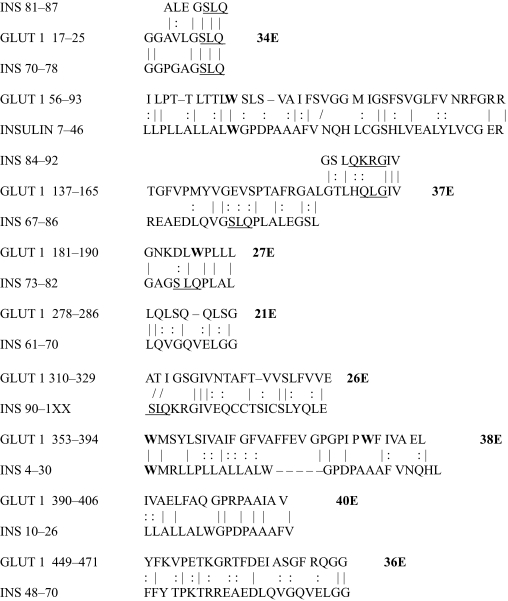
GLUT 1-Insulin similarities.

**Table 2.**
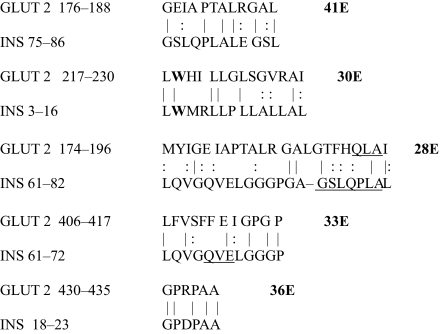
GLUT 2-Insulin similarities.

**Table 3.**
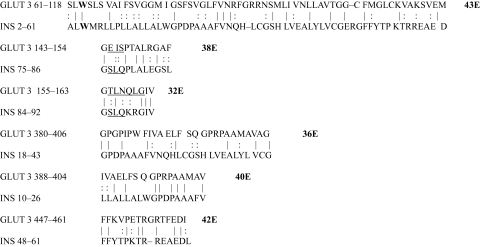
GLUT 3-Insulin similarities.

**Table 4.**
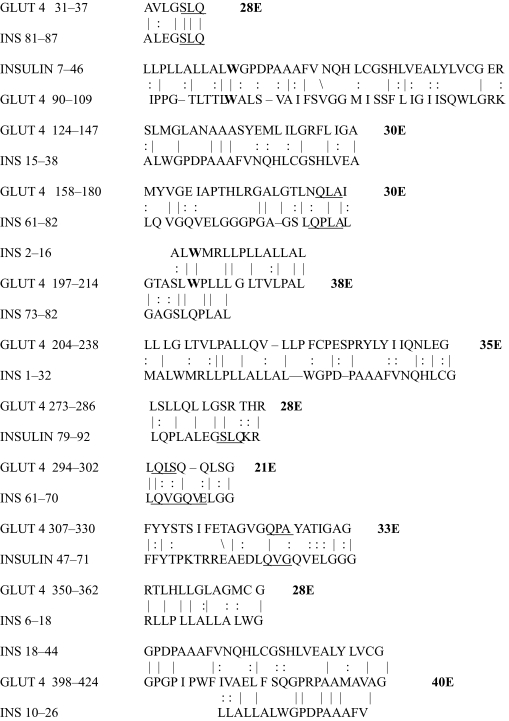
GLUT 4-Insulin similarities.

**Table 5.**
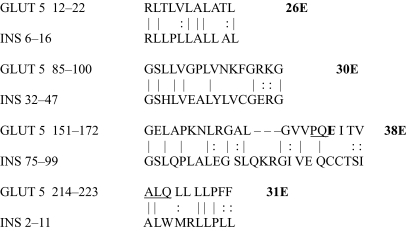
GLUT 5-Insulin similarities.

**Table 6.**
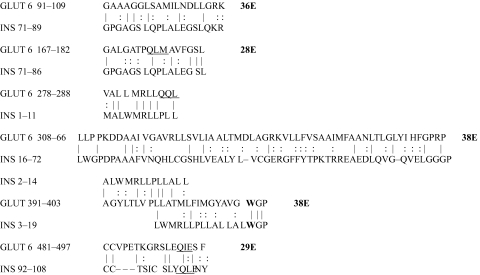
GLUT 6-Insulin similarities.

**Table 7.**
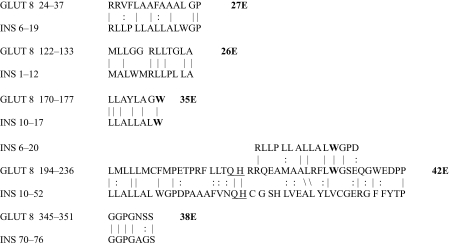
GLUT 8-Insulin similarities.

**Table 8.**
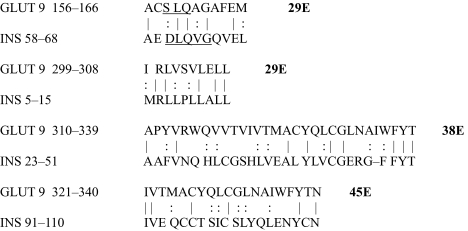
GLUT 9-Insulin similarities.

**Table 9.**
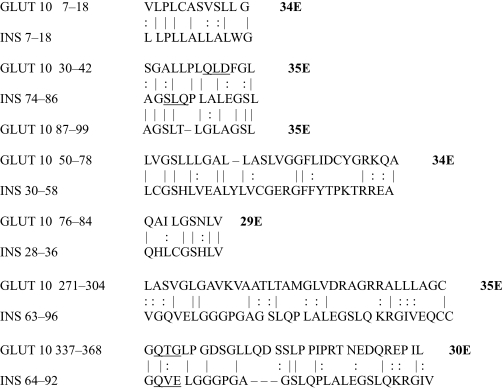
GLUT 10-Insulin similarities.

**Table 10.**
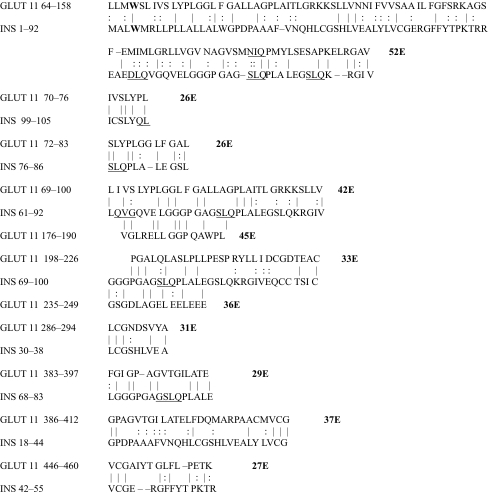
GLUT 11-Insulin similarities.

**Table 11.**
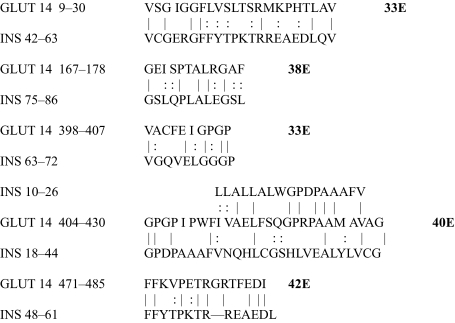
GLUT 14-Insulin similarities.

**Table 12.**
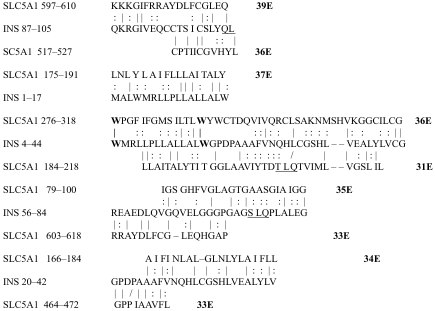
Na+/Glucose high affinity co transporter 1/Insulin similarities.

**Table 13.**
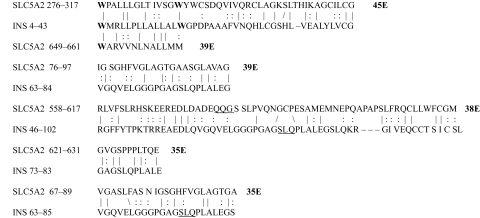
Na+/Glucose low affinity co transporter 2/Insulin similarities.

**Table 14.**
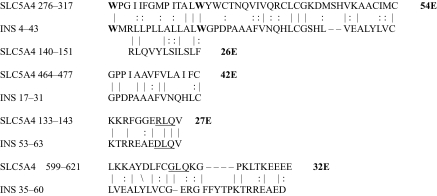
Na+/Glucose low affinity co transporter 3/Insulin similarities.

**Table 15.**
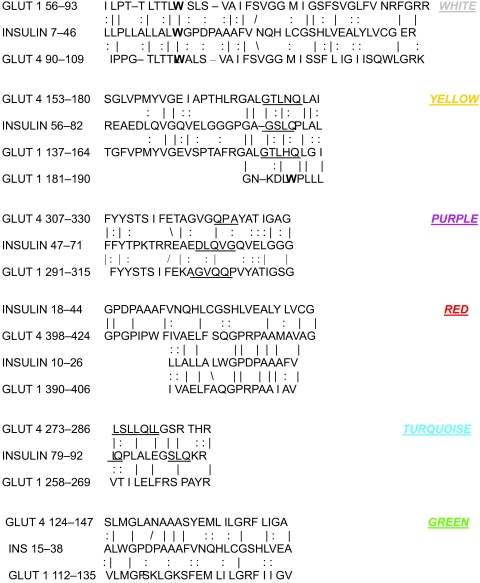
Shared regions of homology between GLUT 1 and GLUT 4 and INSULIN, color coded as the key to Figure 1.
